# 

**Published:** 1886-09

**Authors:** 


### OUR ADVERTISERS.

SEE advertisement of Frederick Stearns & Co., page 28.

THE KENTUCKY SCHOOL OF MEDICINE presents its card to our readers on page 34 of this issue. Young men contemplating a summer course of lectures would do well to correspond with the dean.

R. A. ROBINSON & CO., LOUISVILLE.—No firm in the country manufacture more beautiful or reliable pharmaceutical preparations than this one. Their syrup hypophosphites is equal to any preparation of the kind in the market, and has given general satisfaction in cases in which it has been used here. Their wine of coca is superior to anything of the kind we have seen. See their ad. on page 24 and write for samples.

OUR readers are asked to observe the change in the advertisement of Isaac Phillips, the instrument dealer at 13 N. Broad street, Atlanta. Mr. Phillips has the most complete assortment of surgical instruments and appliances ever brought South, and is selling them at remarkably low prices.

GEO. K. HOPKINS & CO., ST. LOUIS.—These gentlemen are sole agents for the United States and Canada for Stephens' patent saddle-bags and buggy cases. These bags and cases are giving better satisfaction in this section than any ever sold here. They make new quotations in their advertisement on page 33 in this issue of THE JOURNAL.

ACCIDENT INSURANCE.—We desire to call attention to the advertisement of Mr. W. T. Crenshaw in this issue. He represents a first-class Accident Insurance Company which has always paid its losses promptly. A number of heavy losses have recently been paid in this city. A number of Atlanta physicians are insured with him. See the advertisement on page 5, and write him for particulars.

MELLIN'S FOOD.—Prof. Dr. R. Fresenius, of Wiesbaden, Germany, has made an analysis of Mellin's food for infants and invalids, of which the following is a summary:

| | |
|---|---|
| Total carbohydrates | 72.56 |
| Albuminoids | 9.75 |
| Salts | 4.37 |
| Moisture | 13.32 |
| | 100.00 |

Starch and cane sugar, none; reaction alkaline.

A copy of the detailed analysis and remarks of this first chemist in the world may be had by application to Messrs. Doliber, Goodale & Co., Central Wharf, Boston, Mass.

